# COVID-19 vaccination status and factors associated with deaths from the disease, Vitória, 2020-2023: cross-sectional study

**DOI:** 10.1590/S2237-96222025v34e20240700.en

**Published:** 2025-08-08

**Authors:** Tatiane Comerio, João Paulo Cola, Keila Cristina Mascarello, Carolina Maia Martins Sales, Brenda Silva Freire, Adjane da Silva Vasconcelos, Marcia Christina de Souza, Ethel Leonor Noia Maciel

**Affiliations:** 1 Universidade Federal do Espírito Santo, Programa de Pós-Graduação em Saúde Coletiva, Vitória, ES, Brzsil; 2 Secretaria Municipal de Saúde, Vitória, ES, Brazil

**Keywords:** COVID-19, COVID-19 Vaccines, Mortality, Sociodemographic Factors, Comorbidity, Covid-19, Vacunas ontra la Covid-19, Mortalidad, Factores Sociodemográficos, Comorbilidad

## Abstract

**Objective:**

To analyze the vaccination status against COVID-19 and factors associated with deaths from the disease.

**Method:**

Cross-sectional study with secondary data on deaths from COVID-19 in the city of Vitória, Espírito Santo, between February 2020 and December 2023. Individuals under 18 years of age and those with unavailable vaccination status were excluded. Relative and absolute frequencies were calculated by vaccination status. Poisson regression was used to calculate crude and adjusted prevalence ratios (PR) and 95% confidence intervals (95% CI).

**Results:**

A total of 1,460 deaths were included, of which 244 (16.7%) had received ≥2 doses of the vaccines. Among the unvaccinated individuals, the highest frequency of deaths was recorded in individuals aged 60 to 79 years (47.9%), male (56.4%), and non-white race/skin color (53.0%). The PR of death in vaccinated individuals was higher in people ≥80 years old (PR 5.55; 95%CI 2.92; 10.55), 60-79 years old (PR 3.61; 95%CI 1.95; 6.69), with chronic lung disease (PR 1.35; 95%CI 1.03; 1.78) or chronic neurological disease (PR 1.37; 95%CI 1.08; 1.72), respiratory distress (PR 1.51; 95%CI 1.02; 2.23), desaturation (PR 1.97; 95%CI 1.35; 2.88) and adynamia (PR 2.80; 95%CI 2.02; 3.90). People with 1-4 years of schooling (PR 0.63; 95%CI 0.44; 0.90), 5-8 years of schooling (PR 0.57; 95%CI 0.39; 0.84), ≥8 years of schooling (PR 0.66; 95%CI 0.45; 0.97) had a lower prevalence.

**Conclusion:**

The highest prevalence of deaths among vaccinated individuals was associated with age ≥60 years, chronic lung or neurological disease, respiratory distress, desaturation, and adynamia. Loss of immunological memory in the elderly indicates the need for vaccine boosters to prevent deaths.

## Resumen

**Objetivo:**

Analizar el estado de vacunación frente a la covid-19 y los factores asociados a las muertes por la enfermedad.

**Método:**

Estudio transversal con datos secundarios sobre muertes por covid-19 en el municipio de Vitória, Espírito Santo, entre febrero de 2020 y diciembre de 2023. Se excluyeron personas menores de 18 años y aquellas con estado de vacunación no disponible. Se calcularon frecuencias relativas y absolutas según el estado de vacunación. Se utilizó la regresión de Poisson para calcular las razones de prevalencia brutas y ajustadas (RP) y los intervalos de confianza del 95% (IC95%).

**Resultados:**

Se incluyeron 1.460 fallecimientos, de las cuales 244 (16,7%) tenían ≥2 dosis de las vacunas. Entre los no vacunados, la mayor frecuencia de muertes se registró en personas de 60 a 79 años (47,9%), hombres (56,4%), raza/color de piel no blanco (53,0%). La RP de muerte en individuos vacunados fue mayor en personas ≥80 años (RP 5,55; IC95% 2,92; 10,55), 60-79 años (RP 3,61; IC95% 1,95; 6,69), con enfermedad pulmonar crónica (RP 1,35; IC95% 1,03; 1,78) o enfermedad neurológica crónica (RP 1,37; IC95% 1,08; 1,72), dificultad respiratoria (RP 1,51; IC95% 1,02; 2,23), desaturación (RP 1,97; IC95% 1,35; 2,88) y adinamia (RP 2,80; IC95% 2,02; 3,90). Las personas con 1-4 años de escolaridad (RP 0,63; IC95% 0,44; 0,90), 5-8 años de escolaridad (RP 0,57; IC95% 0,39; 0,84), ≥8 años de escolaridad (RP 0,66; IC95% 0,45; 0,97) presentaron una prevalencia menor.

**Conclusión:**

La mayor prevalencia de muertes entre los individuos vacunados se asoció con edad ≥60 años, enfermedad pulmonar o neurológica crónica, dificultad respiratoria, desaturación y adinamia. La pérdida de memoria inmunológica en los ancianos indica la necesidad de refuerzos de vacunas para prevenir muertes.

**Ethical aspects**

This research respected ethical principles, having obtained the following approval data::

Research Ethics Committee: Universidade Federal do Espírito Santo

Opinion number: 4.904.525

Approval date: 8/13/2021

Certificate of Submission for Ethical Appraisal: 49378221.7.0000.5060

Informed Consent Form: Dismissed.

## Introduction

The COVID-19 pandemic, caused by SARS-CoV-2, represented one of the greatest public health challenges in recent history, resulting in millions of deaths worldwide. Even after the implementation of strict control measures and the development of highly effective vaccines, the disease continues to be a significant cause of death in several countries, including Brazil. COVID-19 remains active, with localized outbreaks and new cases, especially in vulnerable groups, such as the elderly and immunocompromised (1).

The scientific race to develop vaccines against COVID-19 was unprecedented, resulting in the approval and global distribution of different vaccines in record time. Studies show that vaccines were highly effective in reducing hospitalization and mortality from COVID-19 (2). However, despite widespread vaccination, there are still significant numbers of deaths related to the disease, and the virus continues to circulate, causing localized outbreaks. In many cases, factors such as advanced age, pre-existing comorbidities and a weakened immune response can influence the clinical outcome, leading to death even in vaccinated individuals (3).

Given this scenario, it is essential to understand the differences between deaths occurring in vaccinated and unvaccinated people. Studies indicate that, while the vaccine protects most individuals, there are subgroups that remain at greater risk of mortality, either due to the presence of multiple comorbidities or the partial ineffectiveness of the immune response (4). On the other hand, among the unvaccinated, mortality is influenced not only by clinical conditions, but also by socioeconomic and demographic factors that hinder access to healthcare and increase exposure to the virus (5,6).

The research sought to identify how sociodemographic and clinical variables influence the vaccination status of individuals who died, providing support for the development of more effective public health strategies to reduce mortality from the disease. In this context, the present study aims to analyze the COVID-19 vaccination status and factors associated with deaths from the disease. 

## Methods

### Study design and context

This is a cross-sectional study using secondary data on deaths from COVID-19 that occurred in Vitória, Espírito Santo. The data were made available by the epidemiological surveillance of the Municipal Health Department.

The municipality of Vitória, capital of the state of Espírito Santo, has an estimated population of 322,869 inhabitants, according to the Brazilian Institute of Geography and Statistics census of 2022 (7). The Municipal Health Department of Vitória, aiming to better reference health actions in the city, stratified its territory into six health regions, namely: Continental region, São Pedro region, Maruípe region, Central region, Santo Antônio region and Forte São João region, whose territorialization is explained in the Municipal Health Plan of the capital.

### Participants

The study included all deaths from COVID-19 of individuals residing in Vitória, occurring between February 2020 and December 2023. Deaths of children under 18 years of age and those without information on vaccination status were excluded.

### Variables and data source

People with COVID-19 were considered to be all those who met the diagnostic criteria of the Ministry of Health (laboratory confirmation, clinical epidemiological criteria) classified in the e-SUS Health Surveillance Information System, the state’s system for reporting diseases and medical conditions.

Deaths from COVID-19 were included for all people with a record in the Mortality Information System, with the underlying cause of death being COVID-19 infection with the following codes, according to the International Classification of Diseases: B34.2 (coronavirus infection unspecified), with the underlying cause being the health condition subsequent to COVID-19 infection, B94.8 (sequelae of other specified infectious and parasitic diseases), and underlying cause of COVID-19 infection in maternal death, O98.5 (other viral diseases complicating pregnancy, childbirth and the puerperium).

Information regarding the type of vaccine, the date of administration of the vaccine, the dose of the vaccine and the identification data of the immunized person were extracted from the Brazilian National Immunization Program Information System (8) and Vacina e Confia (9), the state of Espírito Santo’s own vaccine registration system. 

The databases were paired by identification by full name, date of birth, Individual Taxpayer Registration number and mother’s name. After the automatic pairing, a manual review was performed on a sample of the records to check accuracy and correct possible errors. The process was carried out as part of the service routine during investigations into deaths caused by COVID-19. 

To control bias, data cleaning was performed to standardize vaccination records, eliminating inconsistencies and duplicates. Furthermore, vaccination and mortality data were checked to be sure they were properly paired, to avoid misclassification and to allow death records to be correctly linked to individuals’ vaccination status. 

The dependent variable of the study was vaccination status. Those who had two or more doses registered in the Brazilian National Immunization Program Information System or in the Vacina e Confia system were considered vaccinated.

For deaths confirmation, the Mortality Information System database was used. Covariates were derived from the COVID-19 notification/investigation form and the vaccine registration database, including the following:

a: Sociodemographic:
  - sex (female; male);
  - age group (in years: 18 to 59; 60 to 79; 80 and over);
  - race/skin color (white; non-white);
  - years of education (did not study; 1 to 4; 5 to 8; more than 8; unknown);
  - healthcare professional (yes; no);
  - marital status (married; single; widowed/ divorced);
  - health insurance (private; SUS (Brazilian Unified Health System): exclusive use of SUS).
b. Signs and symptoms:
  - difficulty breathing (yes; no);
  - intercostal retraction (yes; no);
  - desaturation (yes; no);
  - cough (yes; no);
  - sore throat (yes; no);
  - dysphagia (yes; no);
  - diarrhea (yes; no);
  - nausea (yes; no);
  - headache (yes; no);
  - irritability (yes; no);
  - adynamia (yes; no);
  - anosmia (yes; no);
  - dysgeusia (yes; no).
c. Comorbidities:
  - lung disease (yes; no);
  - cardiovascular disease (yes; no);
  - kidney disease (yes; no);
  - liver disease (yes; no);
  - diabetes (yes; no);
  - autoimmune diseases (yes; no);
  - HIV (yes; no);
  - neoplasia (yes; no);
  - smoking (yes; no);
  - bariatric surgery (yes; no);
  - obesity (yes; no);
  - tuberculosis (yes; no);
  - chronic neurological disease (yes; no).

Public institutions were considered to be those managed by the government; and for-profit and philanthropic private institutions were considered to be private, using as a parameter the legal classification of the National Registry of Health Establishments.

### Statistical methods

Relative and absolute frequencies were calculated. Poission regression with robust variance was used to calculate the prevalence ratio (PR). In the raw model, all variables were entered at once. Variables with p-value ≤0.20 were included in the adjusted model, and those with p-value≤0.050 were maintained in the adjusted model. The results of the analyses were expressed in PR, and 95% confidence intervals (95%CI) were estimated. All analyses were performed using Stata v. 14.0 software (StataCorp, College Station, TX, USA). 

## Results

From 2020 to 2023, 1,463 deaths from COVID-19 were recorded in the municipality of Vitória; two deaths of children under 18 years of age were excluded and one death was excluded due to the lack of information on vaccination status. Among the deaths, 1,074 (73.6%) did not have any dose of the COVID-19 vaccine, 244 (16.7%) had two or more doses of the COVID-19 vaccine, and 141 (9.6%) only had one dose of the vaccine.

Among the unvaccinated, the highest frequency of deaths was recorded among those aged 60 to 79 years (47.9%; 95%CI 45.1; 50.7), male (56.4%; 95%CI 53.6; 59.1), non-white race/skin color (53.0%; 95%CI 50.2; 55.8) and those with SUS (Brazilian Unified Health System) health care (55.1%; 95%CI 52.3; 57.9). Among those vaccinated, deaths predominated among those who were aged 80 years or older (53.6%; 95%CI 47.3; 59.8), female (51.2%; 95%CI 44.9; 57.4), white (56.1%; 95%CI 49.8; 62.2) and those who had private health insurance (54.0%; 95%CI 47.7; 60.2). Table 1 shows other characteristics of unvaccinated and vaccinated individuals.

**Table 1:** Absolute (n) and relative (%) frequency with 95% confidence intervals (95%CI) of deaths from COVID-19, by vaccination status, according to sociodemographic variables. Vitória, 2020-2023 (n=1,460)

Variables	Unvaccinated (n=1,216)		Vaccinated (n=244)		
	n	% (IC95%)	n	% (IC95%)	p-value^b^
**Age group** (years)					<0.001
18-59	289	23.7 (21.4; 26.2)	11	4.5 (2.5; 7.9)	
60-79	583	47.9 (45.1; 50.7)	102	41.8 (35.7; 48.1)	
≥80	344	28.2 (25.8; 30.8)	131	53.6 (47.3; 59.8)	
Gender					0.028
Female	530	43.5 (40.8; 46.3)	125	51.2 (44.9; 57.4)	
Male	686	56.4 (53.6; 59.1)	119	48.7 (42.5; 55.0)	
**Race/skin color**					0.009
White	571	46.9 (44.1; 49.7)	137	56.1 (49.8; 62.2)	
Not white	645	53.0 (50.2; 55.8)	107	43.8 (37.7; 50.1)	
**Education** (years)					0.002
No formal education	62	5.0 (3.9; 6.4)	27	11.0 (7.6; 15.6)	
1-4	279	22.9 (20.6; 25.3)	67	27.4 (22.2; 33.4)	
5-8	323	26.5 (24.1; 29.1)	51	20.9 (16.2; 26.4)	
≥8	551	45.3 (42.5; 48.1)	99	40.5 (34.5; 46.8)	
**Health professional**					0.299
Yes	21	1.7 (1.1; 2.6)	2	0.8 (0.2; 3.2)	
No	1,195	98.2 (97.3; 98.8)	242	99.1 (96.7; 99.7)	
**Marital status**					0.001
Married	659	54.2 (51.4; 57.0)	108	38.1 (36.07; 40.24)	
Single	175	14.4 (12.5; 16.4)	28	11.4 (8.0; 16.1)	
Widowed/divorced	381	31.3 (28.8; 34.0)	108	44.2 (38.1; 50.5)	
Agreement					0.008
Private	545	44.8 (42.0; 47.6)	132	54.0 (47.7; 60.2)	
SUS^a^	670	55.1 (52.3; 57.9)	112	45.9 (39.7;52.2)	

^a^ Unified Health System; ^b^ Pearson’s chi-square test.

**Table 2:** Absolute (n) and relative (%) frequency with 95% confidence intervals (95%CI) of deaths from COVID-19, by vaccination status, according to comorbidities. Vitória, 2020-2023 (n=1,460)

Variables	Unvaccinated (n=1,216)		Vaccinated (n=244)		
	n	% (95%CI)	n	% (95%CI)	p-value^a^
Has comorbidity	1,137	93.5 (91.9; 94.7)	235	96.5 (93.0; 98.0)	0.093
Lung disease	158	12.9 (11.2; 15.0)	45	18.4 (14.0; 23.8)	0.025
Cardiovascular disease	922	75.8 (73.3; 78.1)	189	77.4 (71.7; 82.2)	0.584
Kidney disease	97	07.9 (06.5; 09.6)	22	9.0 (6.0;13.3)	0.588
Liver disease	33	02.7 (1.9; 3.7)	5	2.04 (0.8; 4.8)	0.552
Diabetes	552	45.3 (42.6; 48.2)	99	40.5 (34.5; 46.8)	0.167
Immunosuppression	25	2.0 (1.3; 3.0)	3	1.2 (0.3; 3.7)	0.390
HIV	13	1.0 (0.6; 1.8)	2	0.81 (0.2; 3.2)	0.724
Neoplasia	25	2.0 (1.3; 3.0)	9	3.6 (1.9; 6.9)	0.123
Smoking	131	10.7 (9.1; 12.6)	25	10.2 (7.0; 14.7)	0.808
Bariatric surgery	2	0.1 (0.1; 0.6)	0	0	0.526
Obesity	496	40.7 (38.0; 43.5)	73	29.9 (24.4; 35.9)	0.001
Tuberculosis	3	0.2 (0.1; 0.7)	0	0	0.437
Hematological neoplasm	104	8.5 (7.1; 10.2)	27	11.0 (7.6; 15.6)	0.210
Chronic neurological disease	212	17.4 (15.4; 19.6)	79	32.3 (26.7; 38.5)	<0.001

^a^ Pearson’s chi-square test.

**Table 3:** Absolute (n) and relative (%) frequency with intervals 95% confidence interval (95%CI) of deaths from COVID-19, by vaccination status, according to signs and symptoms. Vitória, 2020-2023 (n=1,460)

Variables	Unvaccinated (n=1,216)		Vaccinated (n=244)		
	n	% (95%CI)	n	% (95%CI)	p-value^a^
Fever	882	72.5 (69.9; 74.9)	175	71.7 (65.7; 77.0)	0.796
Respiratory distress	938	77.1 (74.6; 79.4)	218	89.3 (84.7; 92.6)	<0.001
Nasal congestion	25	2.0 (1.3; 3.0)	3	1.2 (0.3; 3.7)	0.39
Intercostal retraction	26	2.1 (1.4;3.1)	2	0.8 (0.2; 3.2)	0.171
Cyanosis	31	2.5 (1.7; 3.6)	9	3.6 (1.9; 6.9)	0.320
Desaturation	835	68.6 (66.0; 71.2)	213	87.2 (82.4; 90.9)	<0.001
Coma	13	1.0 (0.1; 1.8)	1	0.4 (0.1; 2.8)	0.335
Cough	906	74.5 (71.9; 76.8)	200	81.9 (76.6; 86.3)	0.013
Sputum	66	5.4 (4.2; 6.8)	13	5.3 (3.1; 8.9)	0.950
Nasal congestion	178	14.6 (12.7; 16.7)	40	16.3 (12.2; 21.5)	0.483
Nasal discharge	330	27.1 (24.7; 29.7)	66	27.0 (21.8; 32.9)	0.977
Throat	242	19.9 (17.7; 22.2)	34	13.9 (10.1; 18.8)	0.030
Dysphagia	34	2.7 (2.0; 3.8)	3	1.2 (0.3; 3.7)	0.155
Diarrhea	173	14.2 (12.3; 16.3)	244	7.3 (4.6; 11.4)	0.004
Nausea	147	12.0 (10.3; 14.0)	21	8.6 (5.6; 12.8)	0.120
Headache	390	32.0 (29.5; 34.7)	52	21.3 (16.6; 26.9)	0.001
Irritability	37	3.0 (2.2; 4.1)	2	0.8 (0.2; 3.2)	0.049
Adynamia	720	59.2 (56.4; 61.9)	205	84.0 (78.8; 88.1)	<0.001
Exudate	15	1.2 (0.7; 2.0)	2	0.8 (0.2; 3.2)	0.582
Conjunctivitis	4	0.3 (0.1; 0.8)	1	0.4 (0.1; 2.8)	0.844
Convulsion	12	0.9 (0.5;01.7)	3	1.2 (0.3; 3.7)	0.732
Anosmia	138	11.3 (9.6; 13.2)	13	5.3 (3.1; 8.9)	0.005
Dysgeusia	150	12.3 (0.6;14.3)	11	4.5 (2.5; 7.9)	<0.001

^a^ Pearson’s chi-square test.

Clinical characteristics and comorbidities were evaluated, as described in Tables 2 and 3, between unvaccinated and vaccinated individuals. Regarding comorbidities, 1,137 (93.5%; 95%CI 91.9; 94.7) were unvaccinated people, and 235 (96.5%; 95%CI 93.0; 98.0) of those vaccinated had some comorbidity. Obesity (40.7%; 95%CI 38.0; 43.5) was more frequent among the unvaccinated. In turn, among those vaccinated, 45 (18.4%; 95%CI 14.0; 23.8) had lung diseases and 79 (32.3%; 95%CI 26.7; 38.5) had chronic neurological diseases. 

In the raw Poisson model, the prevalence of death among vaccinated individuals was higher in individuals aged 80 years or older (PR 7.52; 95%CI 4.12; 13.68), those with chronic lung disease (PR 1.40; 95%CI 1.05; 1.87), chronic neurological disease (PR 1.92; 95%CI 1.52; 2.43), respiratory distress (PR 2.20; 95%CI 1.50; 3.25), desaturation (PR 2.70; 95%CI 1.89; 3.87), cough (PR 1.45; 95%CI 1.07; 1.97), and adynamia (PR 3.04; 95%CI 2.19; 4.21). In turn, males (PR 0.77; 95%CI 0.62; 0.97), of non-white race/skin color (PR 0.74; 95%CI 0.58; 0.92), with 5 to 8 years of education (PR 0.45; 95%CI 0.29; 0.67), with SUS health care (PR 0.73; 95%CI 0.58; 0.92), with obesity (PR 0.67; 95%CI 0.51; 0.86), with symptoms of sore throat (PR 0.69; 95%CI 0.49; 0.97), headache (PR 0.62; 95%CI 0.47; 0.83), anosmia (PR 0.49; 95%CI 0.29; 0.83) and dysgeusia (PR 0.38; 95%CI 0.21; 0.68) had the lowest prevalence. 

In the adjusted model, the prevalence of death in vaccinated individuals was higher in individuals ≥80 years old (PR 5.55; 95%CI 2.92; 10.55), 60-79 years old (PR 3.61; 95%CI 1.95; 6.69), with chronic lung disease (PR 1.35; 95%CI 1.03; 1.78) or chronic neurological disease (PR 1.37; 95%CI 1.08; 1.72), respiratory distress (PR 1.51; 95%CI 1.02; 2.23), desaturation (PR 1.97; 95%CI 1.35; 2.88) and adynamia (PR 2.80; 95%CI 2.02; 3.90). People with 1-4 years of education (PR 0.63; 95%CI 0.44; 0.90), 5-8 years of schooling (PR 0.57; 95%CI 0.39; 0.84), ≥8 years of schooling (PR 0.66; 95%CI 0.45; 0.97) and headache (PR 0.71; 95%CI 0.54; 0.94) had a lower prevalence (Table 4).

**Table 4:** Raw and adjusted prevalence ratios (PR) and 95% confidence intervals (95%CI) of deaths from COVID-19 among vaccinated people (n=244), by sociodemographic characteristics, comorbidities, and signs and symptoms. Vitória, 2020-2023 (n=1,460)

Variables	Raw analysis		Adjusted analysis	
	PR (95%CI)	p-value^a^	RP (95%CI)	p-value^a^
**Age group** (years)		<0.001		<0.001
18-59	1		1	
60-79	4.06 (2.21; 7.45)		3.61 (1.95; 6.69)	
≥80	7.52 (4.12; 13.68)		5.55 (2.92; 10.55)	
Gender		0.002		0.174
Female	1		1	
Male	0.77 (0.62; 0.97)		0.86 (0.67; 1.10)	
**Race/skin color**		0.004		0.231
White	1		1	
Not white	0.74 (0.58; 0.92)		0.86 (0.68;1.09)	
**Education** (years)		0.009		0.008
No formal education	1		1	
1-4	0.64 (0.43; 0.93)		0.63 (0.44; 0.90)	
5-8	0.45 (0.29; 0.67)		0.57 (0.39; 0.84)	
≥8	0.50 (0.34; 0.72)		0.66 (0.45; 0.97)	
**Marital status**		0.008		0.405
Married	1		1	
Single	0.97 (0.66; 1.44)		1.13 (0.77; 1.66)	
Widowed/divorced	1.57 (1.23; 1.99)		1.07 (0.82; 1.39)	
Agreement		0.008		0.281
Private	1		1	
SUS^b^	0.73 (0.58; 0.92)		0.90 (0.70; 1.15)	
**Has comorbidity**	1.67 (0.89; 3.14)	0.208	-	-
Lung disease	1.40 (1.05; 1.87)	0.021	1.35 (1.03; 1.78)	0.032
Diabetes	0.84 (0.67; 1.07)	0.168	0.86 (0.69; 1.08)	0.076
Neoplasia	1.60 (0.90; 2.84)	0.104	1.47 (0.89; 2.43)	0.187
Obesity	0.67 (0.51; 0.86)	0.001	0.89 (0.69; 1.14)	0.203
Chronic neurological disease	1.92 (1.52; 2.43)	<0.001	1.37 (1.08; 1.72)	0.001
Respiratory distress	2.20 (1.50; 3.25)	<0.001	1.51 (1.02; 2.23)	0.042
**Intercostal retraction**	0.42 (0.11; 1.62)	0.208	-	-
Desaturation	2.70 (1.89; 3.87)	<0.001	1.97 (1.35; 2.88)	<0.001
Cough	1.45 (1.07; 1.97)	0.015	1.34 (0.99; 1.81)	0.108
Sore throat	0.69 (0.49; 0.97)	0.034	0.91 (0.65; 1.26)	0.329
Diarrhea	0.48 (0.16; 1.42)	0.285	-	-
Nausea	0.72 (0.48; 1.01)	0.130	0.80 (0.54; 1.19)	0.477
Headache	0.62 (0.47; 0.83)	0.001	0.71 (0.54; 0.94)	0.010
Irritability	0.30 (0.07; 1.17)	0.286	-	-
Adynamia	3.04 (2.19; 4.21)	<0.001	2.80 (2.02; 3.90)	<0.001
Anosmia	0.49 (0.29; 0.83)	0.008	0.96 (0.52; 1.76)	0.130
Dysgeusia	0.38 (0.21; 0.68)	0.001	0.66 (0.32; 1.37)	0.067

^a^ Pearson’s chi-square test; ^b^ SUS: Unified Health System.

## Discussion

The results of this study show that deaths from COVID-19 differ between vaccinated and unvaccinated individuals, with emphasis on sociodemographic factors and the presence of comorbidities. Among those vaccinated, the prevalence of death was higher in elderly individuals with pulmonary and neurological comorbidities. There is a persistent risk of death from COVID-19 for certain age and sociodemographic groups, even after vaccination.

The introduction of the vaccine in the pandemic scenario was important in reducing hospitalizations and deaths from the disease (10). However, the impact of vaccination on mortality is differentiated by several factors, including age group, comorbidities and sociodemographic conditions (3,10,11). Immunization, despite being an effective tool, does not guarantee total protection among the elderly, partly due to the natural weakening of the immune response in this age group (12). This decline in immunity, combined with the presence of multiple comorbidities, makes the elderly more vulnerable, requiring additional protection strategies, such as continuous vaccination booster doses (3,12-14). 

Variant-adapted vaccines may induce more robust immune responses, offering better protection (15,16). This approach is especially effective in mitigating outbreaks and reducing transmission during periods of high viral circulation. Furthermore, implementing specific strategies aimed at these higher-risk groups is essential to prevent morbidity and mortality in this group. Targeted interventions that consider demographic and social characteristics are also essential, ensuring understanding of the importance of vaccines and prevention measures, in addition to expanding access to health services. 

The fact that women account for a higher proportion of deaths among vaccinated individuals may be related to the general female behavior of seeking health services more frequently. It should be noted that biological sex affects the functions of the immune system, with the sexes having different immune responses, which results in differential susceptibility of men and women to autoimmune diseases, malignancies and infectious diseases, in addition to affecting the outcome of vaccination (17-19). 

While socioeconomic factors may have influenced some aspects of the pandemic, fundamental differences in immune response between men and women are likely a driving factor behind the significant sex bias observed in the COVID-19 pandemic. Even though there is no difference between males and females in terms of the proportion of people infected with SARS-CoV-2, men are at significantly higher risk of severe disease and death than women (17-19).

Chronic lung diseases and chronic neurological diseases were significant factors in death among those vaccinated. These findings are in line with research indicating that severe comorbidities weaken the immune response, increasing the risk of fatal outcomes even in vaccinated individuals (19-21). 

Another relevant factor is obesity, which has been widely recognized as a risk factor for severe forms and mortality from COVID-19 (16,20). However, our result shows a lower prevalence of deaths in this group when vaccinated, which suggests that vaccination can mitigate the impact of this factor. Although obesity increases the risk of complications, vaccination offers significant protection for this risk group (12,13,22).

Even vaccinated individuals may present severe disease symptoms, such as respiratory distress, desaturation and systemic symptoms, such as adynamia (23). This is because vaccines do not completely prevent SARS-CoV-2 infection but rather stimulate the immune system to respond to the infection (23,24). Vaccinated people can still develop severe cases of the disease, especially when faced with more transmissible variants (23,24). Furthermore, individual factors, such as comorbidities (diabetes, hypertension, chronic lung diseases), advanced age and specific individual immune response can influence the manifestation of symptoms (23,24).

Among those who are not vaccinated, there is a higher frequency of deaths in individuals between 60 and 79 years old, male and self-declared black, brown and other races/skin colors. These findings suggest greater vulnerability among racial minority groups, possibly due to lower vaccination coverage and greater exposure to the virus due to social and economic inequalities. Male and ethnic minority populations are at higher risk of mortality from COVID-19 due to lower vaccination adherence and occupational exposure, as well as other factors, such as lack of access to healthcare and worse living conditions, which have a significant impact on mortality from COVID-19, especially in marginalized populations (5,11,20).

The analysis also showed that the highest frequency of death among the unvaccinated occurred among those who did not have private health insurance, indicating that barriers to access to public health may have contributed to the higher mortality. Furthermore, the higher prevalence of deaths among people without education is noteworthy. This disparity is in line with studies indicating that vaccination coverage and access to health care are unequal, especially among low-income, uneducated populations and ethnic minority groups (5.25).

Behavioral and environmental factors also play a significant role in mortality from COVID-19, in addition to the sociodemographic and clinical aspects analyzed. Inadequate adherence to preventive measures, such as mask wearing and social distancing, as well as resistance to vaccination, can increase the risk of exposure to the virus, even among those who are vaccinated. Additionally, factors such as high population density and precarious living conditions, including poorly ventilated environments, intensify the transmission of the virus, increasing mortality rates, even in regions with broad vaccination coverage (26).

This study has some limitations. The use of secondary data may be subject to underreporting or inconsistencies, especially in vaccination and death records. Furthermore, the absence of behavioral variables, such as adherence to preventive measures, and the lack of detailed information on SARS-CoV-2 variants may influence the interpretation of the results. Despite these limitations, the data analyzed provide a robust overview of the characteristics of deaths from COVID-19 in the population studied. 

In conclusion, the highest prevalence of death in vaccinated individuals was associated with age ≥60 years, chronic lung or neurological disease, respiratory distress, desaturation, and adynamia. These findings reinforce the need for targeted strategies to protect the most vulnerable groups. The loss of immunological memory in people over 60 years of age indicates the need for vaccine boosters to provide greater protection against death. The combination of immunization and evidence-based nonpharmacological interventions may help reduce COVID-19 morbidity and mortality in this population.

## Data Availability

The database and analysis codes used in the research will be available upon request from reviewers.
