# Supplementary material for: COVID-19 vaccination status and factors associated with deaths from the disease, Vitória, 2020-2023: cross-sectional study
Source: Epidemiol Serv Saude. 2025 Aug 8;34:e20240700. doi: 10.1590/S2237-96222025v34e20240700.en (PMC12342725; doi:10.1590/S2237-96222025v34e20240700.en)
Supplement: Translation

# Translation: Situação vacinal contra covid-19 e fatores associados aos óbitos pelo agravo, Vitória, 2020-2023: estudo transversal

**DOI:** 10.1590/S2237-96222025v34e20240700.pt

**Authors:** Tatiane Comerio, João Paulo Cola, Keila Cristina Mascarello, Carolina Maia Martins Sales, Brenda Silva Freire, Adjane da Silva Vasconcelos, Marcia Christina de Souza, Ethel Leonor Noia Maciel

**Affiliations:** 1 Universidade Federal do Espírito Santo, Programa de Pós-Graduação em Saúde Coletiva, Vitória, ES, Brasil; 2 Secretaria Municipal de Saúde, Vitória, ES, Brasil

**Keywords:** Covid-19, Vacinas contra Covid-19, Mortalidade, Fatores Sociodemográficos, Comorbidade.

## Abstract

**Objetivo:**

Analisar a situação vacinal contra covid-19 e fatores associados aos óbitos pelo agravo.

**Método:**

Estudo transversal com dados secundários de óbitos por covid-19 do município de Vitória, Espírito Santo, entre fevereiro de 2020 e dezembro de 2023. Foram excluídos menores de 18 anos e situação vacinal indisponível. Calcularam-se frequências relativas e absolutas por situação vacinal. Utilizou-se regressão de Poisson para calcular razões de prevalência (RP) brutas e ajustadas e intervalos de confiança de 95% (IC95%).

**Resultados:**

Incluíram-se 1.460 óbitos, dos quais 244 (16,7%) apresentavam ≥2 doses dos imunizantes. Nos não vacinados, a maior frequência de óbitos foi registrada em pessoas com 60 a 79 anos (47,9%), do sexo masculino (56,4%), raça/cor da pele não branca (53,0%). A RP do óbito nos vacinados foi maior em pessoas ≥80 anos (RP 5,55; IC95% 2,92; 10,55), 60-79 anos (RP 3,61; IC95% 1,95; 6,69), com doença pulmonar crônica (RP 1,35; IC95% 1,03; 1,78) ou neurológica crônica (RP 1,37; IC95% 1,08; 1,72), dificuldade respiratória (RP 1,51; IC95% 1,02; 2,23), dessaturação (RP 1,97; IC95% 1,35; 2,88) e adinamia (RP 2,80; IC95% 2,02; 3,90). Pessoas com 1-4 anos de escolaridade (RP 0,63; IC95% 0,44; 0,90), 5-8 anos de escolaridade (RP 0,57; IC95% 0,39; 0,84), ≥8 anos de escolaridade (RP 0,66; IC95% 0,45; 0,97) apresentaram menor prevalência.

**Conclusão:**

A maior prevalência de óbitos entre os vacinados foi associado a idade ≥60 anos, doença pulmonar ou neurológica crônica, dificuldade respiratória, dessaturação e adinamia. A perda da memória imunológica em idosos indica necessidade de reforços vacinais para prevenir óbitos.

**Aspectos éticos**

A presente pesquisa respeitou os princípios éticos, obtendo os seguintes dados de aprovação: :

Comitê de ética em pesquisa: Universidade Federal do Espírito Santo

Número do parecer: 4.904.525

Data de aprovação: 13/8/2021

Certificado de apresentação de apreciação ética: 49378221.7.0000.5060

Registro do consentimento livre e esclarecido: Dispensado.

## Introdução

A pandemia de covid-19, causada pelo SARS-CoV-2, representou um dos maiores desafios de saúde pública da história recente, resultando em milhões de óbitos em todo o mundo. Mesmo após a implementação de medidas rigorosas de controle e o desenvolvimento de vacinas altamente eficazes, a doença continua a ser uma significativa causa de morte em diversos países, incluindo o Brasil. A covid-19 permanece ativa, com surtos localizados e novos casos, especialmente em grupos vulneráveis, como os idosos e imunocomprometidos (1).

A corrida científica para o desenvolvimento de vacinas contra a covid-19 foi sem precedentes, resultando na aprovação e distribuição global de diferentes imunizantes em tempo recorde. Estudos demonstram que as vacinas foram altamente eficazes em reduzir a hospitalização e a mortalidade por covid-19 (2). No entanto, apesar da vacinação em larga escala, ainda há registros significativos de óbitos relacionados à doença, e o vírus continua a circular, causando surtos localizados. Em muitos casos, fatores como a idade avançada, comorbidades preexistentes e uma resposta imunológica enfraquecida podem influenciar o desfecho clínico, levando à morte mesmo em indivíduos vacinados (3).

Diante deste cenário, torna-se fundamental compreender as diferenças entre os óbitos ocorridos em pessoas vacinadas e não vacinadas. Estudos indicam que, enquanto a vacina protege a maioria dos indivíduos, há subgrupos que permanecem em maior risco de mortalidade, seja pela presença de múltiplas comorbidades ou pela ineficácia parcial da resposta imunológica (4). Por outro lado, entre os não vacinados, a mortalidade é influenciada não apenas pelas condições clínicas, mas também por fatores socioeconômicos e demográficos que dificultam o acesso à saúde e aumentam a exposição ao vírus (5,6).

A pesquisa buscou identificar como variáveis sociodemográficas e clínicas influenciam na situação vacinal de indivíduos que foram a óbito, fornecendo subsídios para o desenvolvimento de estratégias de saúde pública mais eficazes na redução da mortalidade pela doença. Neste contexto, o presente estudo tem como objetivo analisar a situação vacinal contra covid-19 e fatores associados aos óbitos pelo agravo. 

## Métodos

### Delineamento e contexto do estudo

Trata-se de um estudo transversal de dados secundários dos óbitos por covid-19 ocorridos em Vitória, Espírito Santo. Os dados foram disponibilizados pela vigilância epidemiológica da Secretaria Municipal de Saúde.

O município de Vitória, capital do estado do Espírito Santo, segundo o Instituto Brasileiro de Geografia e Estatística de 2022 (7), possui uma população estimada de 322.869 habitantes. A Secretaria Municipal de Saúde de Vitória, visando melhor referenciar as ações em saúde na cidade, estratificou seu território em seis regiões de saúde, a saber: região Continental, região de São Pedro, região de Maruípe, região do Centro, região de Santo Antônio e região do Forte São João, cuja territorialização está explicitada no Plano Municipal de Saúde da capital.

### Participantes

Foram incluídos todos os óbitos por covid-19 de indivíduos residentes em Vitória, ocorridos entre fevereiro de 2020 e dezembro de 2023. Excluíram-se óbitos de menores de 18 anos e aqueles sem informações sobre a situação vacinal.

### Variáveis e fonte de dados

Foram consideradas pessoas com covid-19 todas as que preencheram os critérios diagnósticos do Ministério da Saúde (confirmação laboratorial, critérios clínicos epidemiológicos) classificados no Sistema de Informação em Saúde e-SUS Vigilância em Saúde, sistema próprio estadual de notificação de doenças e agravos.

Foram considerados óbitos por covid-19 todas as pessoas com o registro no Sistema de Informação sobre Mortalidade, com a Classificação Internacional de Doença, de causa básica do óbito com infecção pela covid-19, nas seguintes codificações: B34.2 (infecção pelo coronavírus de localização não especificada), causa básica a condição de saúde posterior a infecção pela covid-19, B94.8 (sequelas de outras doenças infecciosas e parasitárias especificadas), e causa básica infecção pela covid-19 no óbito materno, O98.5 (outras doenças virais complicando a gravidez, o parto e o puerpério).

Informações relativas ao tipo do imunizante, à data de aplicação da vacina, à dose da vacina e aos dados de identificação da pessoa imunizada foram extraídas do Sistema de Informação do Programa Nacional de Imunizações (8) e do Vacina e Confia (9), sistema próprio de registro de vacina do estado do Espírito Santo. 

Foi realizado o pareamento dos bancos de dados através da identificação por nome completo, data de nascimento, número de Cadastro de Pessoa Física e nome da mãe. Após o pareamento automático, uma revisão manual foi realizada em uma amostra dos registros, para verificar a precisão e corrigir possíveis erros. O processo foi realizado na rotina do serviço durante as investigações de óbitos por covid-19. 

Para controle de viés, foi realizada uma limpeza dos dados, a fim de padronizar os registros de vacinas, eliminando inconsistências e duplicatas. Além disso, garantiu-se que os dados vacinais e de mortalidade estivessem devidamente pareados, para evitar erros de classificação e permitir que os registros de óbitos fossem vinculados corretamente à situação vacinal dos indivíduos. 

A variável dependente do estudo foi a situação vacinal. Foram considerados vacinados aqueles que possuíam duas doses ou mais no registro do Sistema de Informação do Programa Nacional de Imunizações ou no sistema Vacina e Confia.

Na confirmação para óbito, foi utilizado o banco do Sistema de Informação sobre Mortalidade. As covariáveis provieram da ficha de notificação/investigação de covid-19 e da base de dados de registro de vacina, incluindo-se as seguintes:

a. Sociodemográficas:
  - sexo (feminino; masculino);
  - faixa etária (em anos: 18 a 59; 60 a 79; 80 e mais);
  - raça/cor da pele (branca; não branca);
  - anos de estudo (não estudou; 1 a 4; 5 a 8; mais de 8; ignorado);
  - profissional de saúde (sim; não);
  - estado civil (casado; solteiro; viúvo/ divorciado);
  - convênio de saúde (privado; Sistema Único de Saúde – SUS: utilização exclusiva do SUS).
b. Sinais e sintomas:
  - dificuldade respiratória (sim; não);
  - tiragem intercostal (sim; não);
  - dessaturação (sim; não);
  - tosse (sim; não);
  - dor de garganta (sim; não);
  - disfagia (sim; não);
  - diarreia (sim; não);
  - náusea (sim; não);
  - cefaleia (sim; não);
  - irritabilidade (sim; não);
  - adinamia (sim; não);
  - anosmia (sim; não);
  - disgeusia (sim; não).
c. Comorbidades:
  - doença pulmonar (sim; não);
  - doença cardiovascular (sim; não);
  - doença renal (sim; não);
  - doença hepática (sim; não);
  - diabetes (sim; não);
  - doenças autoimunes (sim; não);
  - HIV (sim; não);
  - neoplasia (sim; não);
  - tabagismo (sim; não);
  - cirurgia bariátrica (sim; não);
  - obesidade (sim; não);
  - tuberculose (sim; não);
  - doença neurológica crônica (sim; não).

Foram considerados estabelecimentos públicos aqueles geridos pelo poder público; e como privados, estabelecimentos privados com fins lucrativos e filantrópicos, utilizando-se como parâmetro a classificação jurídica da base do Cadastro Nacional de Estabelecimentos de Saúde.

### Métodos estatísticos

Foram calculadas as frequências relativas e absolutas. Utilizou-se a regressão de Poisson de variância robusta para calcular a razão de prevalência (RP). No modelo bruto, foram inseridas todas as variáveis de uma única vez. Foram incluídas no modelo ajustado as variáveis com p-valor≤0,^20^, e foram mantidas no modelo ajustado aquelas que apresentavam p-valor≤0,050. Os resultados das análises foram expressos em RP, e estimaram-se os intervalos de confiança de 95% (IC95%). Todas as análises foram realizadas com utilização do software Stata v. 14.0 (StataCorp, College Station, TX, EUA). 

## Resultados

No período de 2020 a 2023, foram registrados 1.463 óbitos por covid-19 no município de Vitória; dois óbitos de menores de 18 anos foram excluídos e um óbito foi excluído devido à ausência de informação da situação vacinal. Entre os óbitos, 1.074 (73,6%) não apresentavam doses dos imunizantes contra covid-19, 244 (16,7%) apresentavam duas ou mais doses de imunizantes contra covid-19, e 141 (9,6%) somente uma dose de imunizante.

Entre os não vacinados, a maior frequência de óbitos foi registrada entre os que estavam na faixa etária de 60 a 79 anos (47,9%; IC95% 45,1; 50,^7^), do sexo masculino (56,4%; IC95% 53,6; 59,^1^), raça/cor da pele não branca (53,0%; IC95% 50,2; 55,^8^) e os que possuíam convênio de saúde SUS (55,1%; IC95% 52,3; 57,^9^). Já entre os vacinados, predominaram os óbitos naqueles que estavam na faixa etária maior ou igual a 80 anos (53,6%; IC95% 47,3; 59,^8^), do sexo feminino (51,2%; IC95% 44,9; 57,^4^), de raça/cor da pele branca (56,1%; IC95% 49,8; 62,^2^) e os que possuíam convênio de saúde privado (54,0%; IC95% 47,7; 60,^2^). As demais características dos não vacinados e dos vacinados estão descritas na Tabela 1.

**Tabela 1:** Frequência absoluta (n) e relativa (%) com intervalos de confiança de 95% (IC95%) dos óbitos por covid-19, por situação vacinal, segundo variáveis sociodemográficas. Vitória, 2020-2023 (n=1.460)

Variáveis	Não vacinado (n=1.216)		Vacinado (n=244)		
	n	% (IC95%)	n	% (IC95%)	p-valor ^b^
**Faixa etária** (anos)					<0,001
18-59	289	23,7 (21,4; 26,2)	11	4,5 (2,5; 7,9)	
60-79	583	47,9 (45,1; 50,7)	102	41,8 (35,7; 48,1)	
≥80	344	28,2 (25,8; 30,8)	131	53,6 (47,3; 59,8)	
Sexo					0,028
Feminino	530	43,5 (40,8; 46,3)	125	51,2 (44,9; 57,4)	
Masculino	686	56,4 (53,6; 59,1)	119	48,7 (42,5; 55,0)	
**Raça/cor da pele**					0,009
Branca	571	46,9 (44,1; 49,7)	137	56,1 (49,8; 62,2)	
Não branca	645	53,0 (50,2; 55,8)	107	43,8 (37,7; 50,1)	
**Escolaridade** (anos)					0,002
Sem estudo	62	5,0 (3,9; 6,4)	27	11,0 (7,6; 15,6)	
1-4	279	22,9 (20,6; 25,3)	67	27,4 (22,2; 33,4)	
5-8	323	26,5 (24,1; 29,1)	51	20,9 (16,2; 26,4)	
≥8	551	45,3 (42,5; 48,1)	99	40,5 (34,5; 46,8)	
**Profissional de saúde**					0,299
Sim	21	1,7 (1,1; 2,6)	2	0,8 (0,2; 3,2)	
Não	1.195	98,2 (97,3; 98,8)	242	99,1 (96,7; 99,7)	
**Estado civil**					0,001
Casado	659	54,2 (51,4; 57,0)	108	44,2 (38,1; 50,5)	
Solteiro	175	14,4 (12,5; 16,4)	28	11,4 (8,0; 16,1)	
Viúvo/ divorciado	381	31,3 (28,8; 34,0)	108	44,2 (38,1; 50,5)	
Convênio					0,008
Privado	545	44,8 (42,0; 47,6)	132	54,0 (47,7; 60,2)	
SUS^a^	670	55,1 (52,3; 57,9)	112	45,9 (39,7;52,2)	

^a^ Sistema Único de Saúde; ^b^ Teste qui-quadrado de Pearson.

**Tabela 2:** Frequência absoluta (n) e relativa (%) com intervalos de confiança de 95% (IC95%) dos óbitos por covid-19, por situação vacinal, segundo comorbidades. Vitória, 2020-2023 (n=1.460)

Variáveis	Não vacinado (n=1.216)		Vacinado (n=244)		
	n	% (IC95%)	n	% (IC95%)	p-valor^a^
Possui comorbidade	1.137	93,5 (91,9; 94,7)	235	96,5 (93,0; 98,0)	0,093
Doença pulmonar	158	12,9 (11,2; 15,0)	45	18,4 (14,0; 23,8)	0,025
Doença cardiovascular	922	75,8 (73,3; 78,1)	189	77,4 (71,7; 82,2)	0,584
Doença renal	97	07,9 (06,5; 09,6)	22	9,0 (6,0;13,3)	0,588
Doença hepática	33	02,7 (1,9; 3,7)	5	2,04 (0,8; 4,8)	0,552
Diabetes	552	45,3 (42,6; 48,2)	99	40,5 (34,5; 46,8)	0,167
Imunossupressão	25	2,0 (1,3; 3,0)	3	1,2 (0,3; 3,7)	0,390
HIV	13	1,0 (0,6; 1,8)	2	0,81 (0,2; 3,2)	0,724
Neoplasia	25	2,0 (1,3; 3,0)	9	3,6 (1,9; 6,9)	0,123
Tabagismo	131	10,7 (9,1; 12,6)	25	10,2 (7,0; 14,7)	0,808
Cirurgia bariátrica	2	0,1 (0,1; 0,6)	0	0	0,526
Obesidade	496	40,7 (38,0; 43,5)	73	29,9 (24,4; 35,9)	0,001
Tuberculose	3	0,2 (0,1; 0,7)	0	0	0,437
Neoplasia hematológica	104	8,5 (7,1; 10,2)	27	11,0 (7,6; 15,6)	0,210
Doença neurológica crônica	212	17,4 (15,4; 19,6)	79	32,3 (26,7; 38,5)	<0,001

^a^ Teste qui-quadrado de Pearson.

**Tabela 3:** Frequência absoluta (n) e relativa (%) com intervalos de confiança de 95% (IC95%) dos óbitos por covid-19, por situação vacinal, segundo sinais e sintomas. Vitória, 2020-2023 (n=1.460)

Variáveis	Não vacinado (n=1.216)		Vacinado (n=244)		
	n	% (IC95%)	n	% (IC95%)	p-valor^a^
Febre	882	72,5 (69,9; 74,9)	175	71,7 (65,7; 77,0)	0,796
Dificuldade respiratória	938	77,1 (74,6; 79,4)	218	89,3 (84,7; 92,6)	<0,001
Congestão nasal	25	2,0 (1,3; 3,0)	3	1,2 (0,3; 3,7)	0,39
Tiragem intercostal	26	2,1 (1,4;3,1)	2	0,8 (0,2; 3,2)	0,171
Cianose	31	2,5 (1,7; 3,6)	9	3,6 (1,9; 6,9)	0,320
Dessaturação	835	68,6 (66,0; 71,2)	213	87,2 (82,4; 90,9)	<0,001
Coma	13	1,0 (0,1; 1,8)	1	0,4 (0,1; 2,8)	0,335
Tosse	906	74,5 (71,9; 76,8)	200	81,9 (76,6; 86,3)	0,013
Escarro	66	5,4 (4,2; 6,8)	13	5,3 (3,1; 8,9)	0,950
Congestão nasal	178	14,6 (12,7; 16,7)	40	16,3 (12,2; 21,5)	0,483
Coriza	330	27,1 (24,7; 29,7)	66	27,0 (21,8; 32,9)	0,977
Garganta	242	19,9 (17,7; 22,2)	34	13,9 (10,1; 18,8)	0,030
Disfagia	34	2,7 (2,0; 3,8)	3	1,2 (0,3; 3,7)	0,155
Diarreia	173	14,2 (12,3; 16,3)	244	7,3 (4,6; 11,4)	0,004
Náusea	147	12,0 (10,3; 14,0)	21	8,6 (5,6; 12,8)	0,120
Cefaleia	390	32,0 (29,5; 34,7)	52	21,3 (16,6; 26,9)	0,001
Irritabilidade	37	3,0 (2,2; 4,1)	2	0,8 (0,2; 3,2)	0,049
Adinamia	720	59,2 (56,4; 61,9)	205	84,0 (78,8; 88,1)	<0,001
Exsudato	15	1,2 (0,7; 2,0)	2	0,8 (0,2; 3,2)	0,582
Conjuntivite	4	0,3 (0,1; 0,8)	1	0,4 (0,1; 2,8)	0,844
Convulsão	12	0,9 (0,5;01,7)	3	1,2 (0,3; 3,7)	0,732
Anosmia	138	11,3 (9,6; 13,2)	13	5,3 (3,1; 8,9)	0,005
Disgeusia	150	12,3 (0,6;14,3)	11	4,5 (2,5; 7,9)	<0,001

^a^ Teste qui-quadrado de Pearson.

As características clínicas e as comorbidades foram avaliadas, conforme descrito nas tabelas 2 e 3, entre os não vacinados e os vacinados. Quanto às comorbidades, 1.137 (93,5%; IC95% 91,9; 94,^7^) eram das pessoas não vacinadas, e 235 (96,5%; IC95% 93,0; 98,0) das vacinadas possuíam alguma comorbidade. A obesidade (40,7%; IC95% 38,0; 43,^5^) foi mais frequente entre os não vacinados. Por sua vez, entre os vacinados, 45 (18,4%; IC95% 14,0; 23,^8^) eram portadores de doenças pulmonares e 79 (32,3%; IC95% 26,7;38,^5^) de doença neurológica crônica. 

No modelo de Poisson bruto, a prevalência do óbito entre os vacinados foi maior nas pessoas com faixa etária igual ou superior a 80 anos (RP 7,52; IC95% 4,12; 13,68), portadores de doença pulmonar crônica (RP 1,40; IC95% 1,05; 1,87), doença neurológica crônica (RP 1,92; IC95% 1,52; 2,43), dificuldade respiratória (RP 2,20; IC95% 1,50; 3,^25^), dessaturação (RP 2,70; IC95% 1,89; 3,87), tosse (RP 1,45; IC95% 1,07; 1,97) e adinamia (RP 3,04; IC95% 2,19; 4,^21^). Por seu turno, os do sexo masculino (RP 0,77; IC95% 0,62; 0,97), de raça/cor da pele não branca (RP 0,74; IC95% 0,58; 0,92), com 5 a 8 anos de estudo (RP 0,45; IC95% 0,29; 0,67), com convênio de saúde SUS (RP 0,73; IC95% 0,58; 0,92), com obesidade (RP 0,67; IC95% 0,51; 0,86), com sintomas de dor de garganta (RP 0,69; IC95% 0,49; 0,97), cefaleia (RP 0,62; IC95% 0,47; 0,83), anosmia (RP 0,49; IC95% 0,29; 0,83) e disgeusia (RP 0,38; IC95% 0,21; 0,68) apresentaram a menor prevalência. 

No modelo ajustado, A prevalência do óbito nos vacinados foi maior em pessoas ≥80 anos (RP 5,55; IC95% 2,92; 10,55), 60-79 anos (RP 3,61; IC95% 1,95; 6,69), com doença pulmonar crônica (RP 1,35; IC95% 1,03; 1,78) ou neurológica crônica (RP 1,37; IC95% 1,08; 1,72), dificuldade respiratória (RP 1,51; IC95% 1,02; 2,^23^), dessaturação (RP 1,97; IC95% 1,35; 2,88) e adinamia (RP 2,80; IC95% 2,02; 3,90). Pessoas com 1-4 anos de escolaridade (RP 0,63; IC95% 0,44; 0,90), 5-8 anos de escolaridade (RP 0,57; IC95% 0,39; 0,84), ≥8 anos de escolaridade (RP 0,66; IC95% 0,45; 0,97) e cefaleia (RP 0,71; IC95% 0,54; 0,94) apresentaram menor prevalência (Tabela 4).

**Tabela 4:** Razões de prevalência (RP) bruta e ajustada, e intervalos de confiança de 95% (IC95%) dos óbitos por covid-19, de pessoas vacinadas (n=244), pelas características sociodemográficas, comorbidades e sinais e sintomas. Vitória, 2020-2023 (n=1.460)

Variáveis	Análise bruta		Análise ajustada	
	RP (IC95%)	p-valor^a^	RP (IC95%)	p-valor^a^
**Faixa etária** (anos)		<0,001		<0,001
18-59	1		1	
60-79	4,06 (2,21; 7,45)		3,61 (1,95; 6,69)	
≥80	7,52 (4,12; 13,68)		5,55 (2,92; 10,55)	
Sexo		0,002		0,174
Feminino	1		1	
Masculino	0,77 (0,62; 0,97)		0,86 (0,67; 1,10)	
**Raça/cor da pele**		0,004		0,231
Branca	1		1	
Não branca	0,74 (0,58; 0,92)		0,86 (0,68;1 ,09)	
**Escolaridade** (anos)		0,009		0,008
Sem estudo	1		1	
1-4	0,64 (0,43; 0,93)		0,63 (0,44; 0,90)	
5-8	0,45 (0,29; 0,67)		0,57 (0,39; 0,84)	
≥8	0,50 (0,34; 0,72)		0,66 (0,45; 0,97)	
**Estado civil**		0,008		0,405
Casado	1		1	
Solteiro	0,97 (0,66; 1,44)		1,13 (0,77; 1,66)	
Viúvo/divorciado	1,57 (1,23; 1,99)		1,07 (0,82; 1,39)	
Convênio		0,008		0,281
Privado	1		1	
SUS^b^	0,73 (0,58; 0,92)		0,90 (0,70; 1,15)	
**Possui comorbidade**	1,67 (0,89; 3,14)	0,208	-	-
Doença pulmonar	1,40 (1,05; 1,87)	0,021	1,35 (1,03; 1,78)	0,032
Diabetes	0,84 (0,67; 1,07)	0,168	0,86 (0,69; 1,08)	0,076
Neoplasia	1,60 (0,90; 2,84)	0,104	1,47 (0,89; 2,43)	0,187
Obesidade	0,67 (0,51; 0,86)	0,001	0,89 (0,69; 1,14)	0,203
Doença neurológica crônica	1,92 (1,52; 2,43)	<0,001	1,37 (1,08; 1,72)	0,001
Dificuldade respiratória	2,20 (1,50; 3,25)	<0,001	1,51 (1,02; 2,23)	0,042
**Tiragem intercostal**	0,42 (0,11; 1,62)	0,208	-	-
Dessaturação	2,70 (1,89; 3,87)	<0,001	1,97 (1,35; 2,88)	<0,001
Tosse	1,45 (1,07; 1,97)	0,015	1,34 (0,99; 1,81)	0,108
Dor de garganta	0,69 (0,49; 0,97)	0,034	0,91 (0,65; 1,26)	0,329
Diarreia	0,48 (0,16; 1,42)	0,285	-	-
Náusea	0,72 (0,48; 1,01)	0,130	0,80 (0,54; 1,19)	0,477
Cefaleia	0,62 (0,47; 0,83)	0,001	0,71 (0,54; 0,94)	0,010
Irritabilidade	0,30 (0,07; 1,17)	0,286	-	-
Adinamia	3,04 (2,19; 4,21)	<0,001	2,80 (2,02; 3,90)	<0,001
Anosmia	0,49 (0,29; 0,83)	0,008	0,96 (0,52; 1,76)	0,130
Disgeusia	0,38 (0,21; 0,68)	0,001	0,66 (0,32; 1,37)	0,067

^a^ Teste qui-quadrado de Pearson; ^b^ SUS: Sistema Único de Saúde.

## Discussão

Os resultados deste estudo evidenciam que os óbitos por covid-19 apresentam diferenças entre indivíduos vacinados e não vacinados, destacando-se os fatores sociodemográficos e a presença de comorbidades. Entre os vacinados, a prevalência do óbito foi maior em indivíduos idosos, portadores de comorbidades de tipo pulmonar e neurológica. Observa-se a persistência de riscos para óbitos por covid-19 para determinados grupos etários e sociodemográficos, mesmo após a vacinação.

A introdução da vacina no cenário da pandemia foi importante para a redução de hospitalizações e mortes pela doença (10). No entanto, o impacto da vacinação na mortalidade é diferenciado por diversos fatores, incluindo-se faixa etária, comorbidades e condições sociodemográficas (3,10,11). A imunização, apesar de ser uma ferramenta eficaz, não garante proteção total entre os idosos, em parte devido ao enfraquecimento natural da resposta imunológica nessa faixa etária (12). Esse declínio da imunidade, combinado com a presença de múltiplas comorbidades, torna os idosos mais vulneráveis, exigindo estratégias de proteção adicionais, como reforço vacinal contínuo (3,12-14). 

Vacinas adaptadas para variantes podem induzir respostas imunológicas mais robustas, oferecendo melhor proteção (15,16). Essa abordagem é especialmente eficaz para mitigar surtos e reduzir a transmissão em períodos de alta circulação viral. Para além disso, implementar estratégias específicas voltadas para esses grupos de maior risco é essencial para evitar a morbimortalidade neste grupo. Também são fundamentais intervenções direcionadas que considerem as características demográficas e sociais, que garantam a compreensão da importância das vacinas e das medidas de prevenção, além da ampliação do acesso a serviços de saúde. 

O fato de pessoas do sexo feminino apresentarem uma maior proporção entre os óbitos de vacinados pode estar relacionado ao comportamento feminino, de modo geral, em buscar com maior frequência os serviços de saúde. Observe-se que o sexo biológico afeta as funções do sistema imunológico, tendo os sexos respostas imunes diferentes, o que resulta em suscetibilidade diferencial de homens e mulheres a doenças autoimunes, malignidades e doenças infecciosas, além de afetar o resultado da vacinação (17-19). 

Embora fatores socioeconômicos possam ter influenciado alguns aspectos da pandemia, as diferenças fundamentais na resposta imune entre homens e mulheres provavelmente são um fator determinante por trás do viés sexual significativo observado na pandemia de covid-19. Mesmo que não haja diferença de sexo na proporção de pessoas infectadas com SARS-CoV-2, os homens correm um risco significativamente maior de doença grave e morte do que as mulheres (17-19).

Doenças pulmonares crônicas e doenças neurológicas crônicas foram fatores significativos para o óbito entre os vacinados. Esses achados estão alinhados com pesquisas que indicam que comorbidades graves enfraquecem a resposta imunológica, aumentando o risco de desfechos fatais mesmo em indivíduos vacinados (19-21). 

Outro fator relevante é a obesidade, que tem sido amplamente reconhecida como um fator de risco para formas graves e mortalidade de covid-19 (16,20). No entanto, nosso resultado apresenta uma menor prevalência de óbitos nesse grupo quando vacinado, o que sugere que a vacinação pode mitigar o impacto desse fator. Embora a obesidade aumente o risco de complicações, a vacinação oferece proteção significativa para esse grupo de risco (12,13,22).

Mesmo indivíduos vacinados podem apresentar sintomas de gravidade da doença, como dificuldade respiratória, dessaturação e sintomas sistêmicos, como adinamia (23). Isso ocorre porque as vacinas não impedem completamente a infecção pelo SARS-CoV-2, mas estimulam o sistema imunológico a responder à infecção (23,24). Pessoas vacinadas ainda podem desenvolver quadros graves da doença, especialmente diante de variantes mais transmissíveis (23,24). Além disso, fatores individuais, como comorbidades (diabetes, hipertensão, doenças pulmonares crônicas), idade avançada e resposta imunológica específica de cada pessoa podem influenciar na manifestação dos sintomas (23,24).

Entre os não vacinados, há uma maior frequência de óbitos em indivíduos entre 60 e 79 anos, do sexo masculino e autodeclarados pretos, pardos e outras raças/cores da pele. Estes achados sugerem uma vulnerabilidade maior entre grupos racialmente minoritários, possivelmente devido à menor cobertura vacinal e à maior exposição ao vírus em função de desigualdades sociais e econômicas. As populações masculina e de minorias étnicas têm maior risco de mortalidade por covid-19 devido à menor adesão à vacinação e à exposição ocupacional, como também a outros fatores, como acesso à saúde e piores condições de vida, que possuem impacto significativo na mortalidade por covid-19, especialmente em populações marginalizadas (5,11,20).

A análise também mostrou que a maior frequência do óbito entre os não vacinados ocorreu entre os que não tinham convênio de saúde privado, indicando que barreiras ao acesso à saúde pública podem ter contribuído para a mortalidade mais alta. Destaca-se, além disso, a maior prevalência de óbitos em pessoas sem escolaridade. Essa disparidade está alinhada com estudos que indicam que a cobertura vacinal e o acesso a cuidados de saúde são desiguais, especialmente entre populações de baixa renda, sem escolaridade e grupos étnicos minoritários (5,25).

Fatores comportamentais e ambientais também desempenham um papel significativo na mortalidade por covid-19, além dos aspectos sociodemográficos e clínicos analisados. A adesão inadequada às medidas preventivas, como o uso de máscaras e o distanciamento social, bem como a resistência à vacinação, pode aumentar o risco de exposição ao vírus, mesmo entre os vacinados. Adicionalmente, fatores como alta densidade populacional e condições de vida precárias, incluindo ambientes mal ventilados, intensificam a transmissão do vírus, elevando as taxas de mortalidade, mesmo em regiões com ampla cobertura vacinal (26).

O estudo apresenta algumas limitações. O uso de dados secundários pode estar sujeito a subnotificações ou inconsistências, especialmente nos registros de vacinação e óbitos. Além disso, a ausência de variáveis comportamentais, como adesão a medidas preventivas, e a falta de informações detalhadas sobre as variantes do SARS-CoV-2 podem influenciar a interpretação dos resultados. Apesar dessas limitações, os dados analisados fornecem um panorama robusto das características dos óbitos por covid-19 na população estudada. 

Em conclusão, a maior prevalência do óbito nos vacinados foi associada a idade ≥60 anos, doença pulmonar ou neurológica crônica, dificuldade respiratória, dessaturação e adinamia. Esses achados reforçam a necessidade de estratégias direcionadas para proteger os grupos mais vulneráveis. A perda da memória imunológica em pessoas acima de 60 anos indica necessidade de reforços vacinais para maior proteção contra óbito. A combinação de imunização e intervenções não farmacológicas baseadas em evidências pode ajudar a reduzir a morbimortalidade da covid-19 nessa população.

## Data Availability

O banco de dados e os códigos de análise utilizados na pesquisa estarão disponíveis sob demanda dos pareceristas.
